# Severe Hypertriglyceridemia-Induced Pancreatitis Presenting With Diabetic Ketoacidosis in a Pediatric Patient

**DOI:** 10.7759/cureus.99289

**Published:** 2025-12-15

**Authors:** Angeles L Padilla, Laura Davila Parrilla, Miguel F Agrait Gonzalez, Acela Rosado, Glorimar Salcedo

**Affiliations:** 1 Emergency Medicine, Centro Médico Episcopal San Lucas, Ponce, PRI; 2 Emergency Medicine, Ponce Health Sciences University, Ponce, PRI; 3 Rheumatology, Manning Family Children's Hospital, New Orleans, USA; 4 Pediatrics, Puerto Rico Women and Children's Hospital, Bayamon, USA

**Keywords:** de novo diabetes mellitus, diabetic ketoacidosis, familial hypertriglyceridemia, hypertriglyceridemia induced pancreatitis, lipemic serum, pediatric pancreatitis, xanthelasma

## Abstract

Hypertriglyceridemia (HTG) is a recognized cause of acute pancreatitis (AP) in adults, but it remains an uncommon etiology in pediatric patients, particularly when occurring concurrently with new-onset diabetes mellitus (DM) and diabetic ketoacidosis (DKA). We present the case of a previously healthy 12-year-old female who developed severe hypertriglyceridemia-induced acute pancreatitis with simultaneous DKA at initial presentation of type 1 diabetes. The patient arrived at the emergency department with acute epigastric pain, emesis, diarrhea, and severe dehydration. Physical examination revealed xanthelasma and xanthomas, while laboratory testing showed markedly lipemic serum with triglycerides of 15,630 mg/dL, cholesterol of 561 mg/dL, and HbA1c of 10.4%. She was admitted to the pediatric intensive care unit for management of DKA, multiorgan dysfunction, and pancreatitis. Treatment included aggressive intravenous fluid resuscitation, electrolyte correction, and continuous insulin infusion followed by transition to subcutaneous insulin. Over nine days, her triglyceride levels normalized, pancreatitis resolved, and renal function recovered. She was discharged on a diabetic diet, insulin regimen, and lipid-lowering therapy with olezarsen for familial hypertriglyceridemia. Two years post-diagnosis, her diabetes and lipid levels remain well controlled.

This case underscores the diagnostic challenge of acute pancreatitis in pediatric DKA, where overlapping abdominal symptoms may obscure the underlying etiology. The lipemic appearance of blood samples can serve as an important bedside clue to severe hypertriglyceridemia. Recognition of this association is critical, as prompt insulin therapy can simultaneously address both DKA and triglyceride reduction. Clinicians should maintain a high index of suspicion for HTG-induced pancreatitis in children presenting with DKA, particularly when serum appears lipemic, and should investigate for underlying familial dyslipidemia. Early identification and comprehensive management, encompassing fluid and electrolyte balance, metabolic stabilization, and long-term lipid control, are key to preventing recurrence and reducing morbidity in this rare but serious presentation.

## Introduction

Hypertriglyceridemia (HTG) is the third most common cause of acute pancreatitis (AP) in adults [1]. Hypertriglyceridemia as a risk factor for pancreatitis is generally accepted as serum triglycerides (TGs) greater than 1000 mg/dL, with studies showing a positive correlation between high TGs and the severity of AP [1,2]. While HTG in the setting of newly diagnosed diabetes mellitus (DM) is often seen in both adults and pediatric patients, its incidence as the etiology for pancreatitis leading to diabetic ketoacidosis has scarcely been studied in pediatric patients compared to adults [3]. We discuss a previously healthy 12-year-old female who presented to the ED with severe hypertriglyceridemia-induced acute pancreatitis and diabetic ketoacidosis. Our aim is to highlight the importance of screening for hypertriglyceridemia as a possible etiology for pediatric pancreatitis and a possible inciting factor for DKA.

## Case presentation

A 12-year-old previously healthy female with no past medical history was brought in for the acute onset of abdominal pain that began that morning. She described the pain as sharp with tenderness in the epigastric region, accompanied by loose stools and multiple episodes of non-bloody, non-bilious emesis. Symptoms were accompanied by general malaise and dizziness. Further history revealed polyuria, polydipsia, polyphagia, weight loss, and intermittent episodes of emesis and diarrhea, ongoing for at least one to two weeks. Amenorrhea was reported for the past three months. Parents reported the patient usually followed a balanced diet, including fruits and vegetables. They described no previous similar episodes, any sick contacts, or any family history of similar issues.

**Figure 1:**
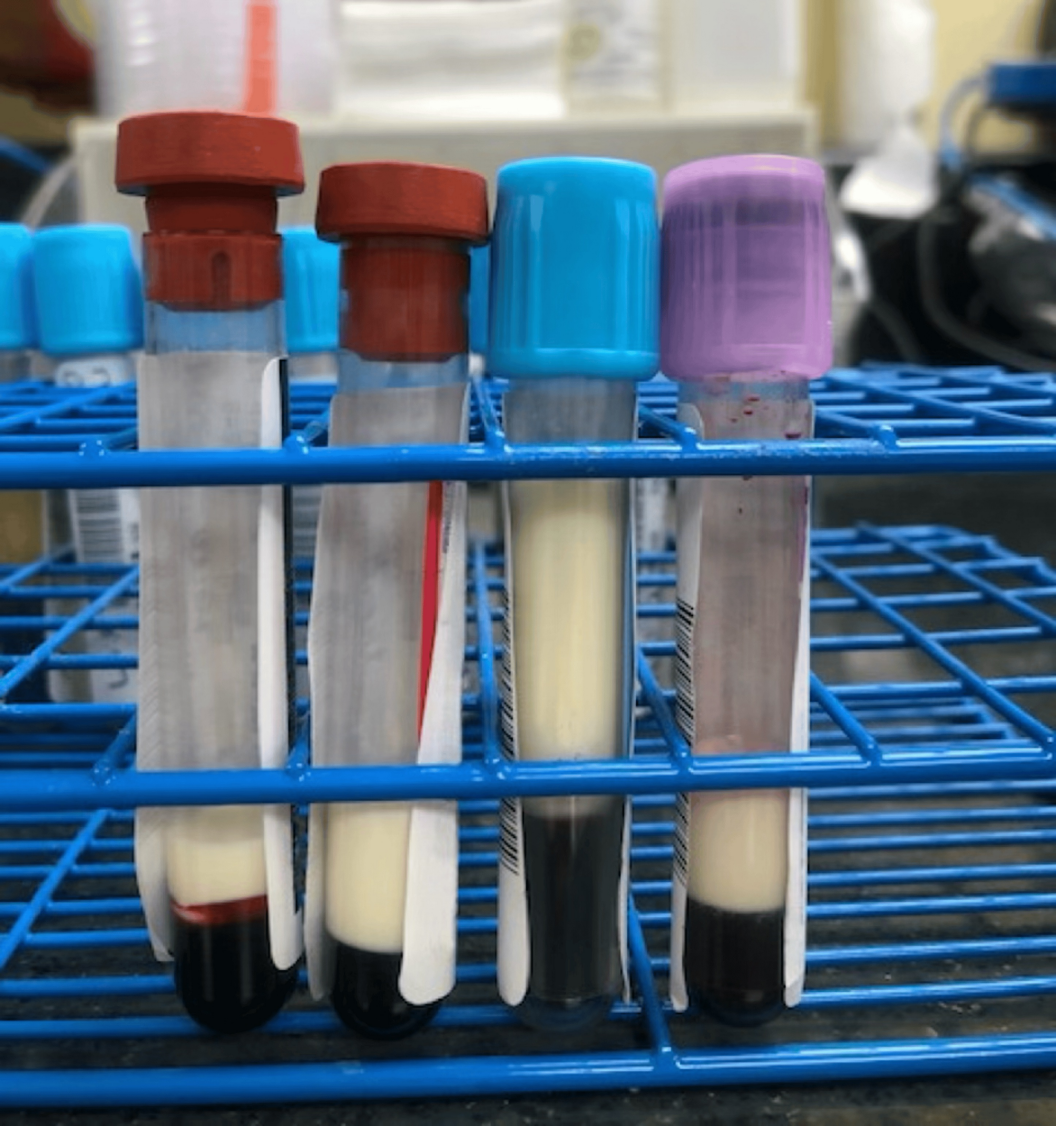
Lipemic blood samples were obtained from the patient. This was their appearance shortly after blood draw and without centrifugation.

A physical exam found sunken eyes, dry mucosa, cracked lips, and capillary refill >3 seconds, all consistent with dehydration. Further evaluation revealed epigastric abdominal pain, xanthelasma of the eyelids, and xanthomas distributed around the neck. Blood drawn was observed as abundantly lipemic (Figure 1). Initial laboratory reports suggested diabetic ketoacidosis (DKA), diabetes de novo (DM), acute pancreatitis, and hypertriglyceridemia with a value of 15,630 mg/dL.

The patient was admitted to the PICU for close monitoring and treatment of type 1 DM, severe dehydration, multiorgan system dysfunction, dyslipidemia, pancreatitis, and acute kidney injury. The patient had episodes of hyponatremia, hypokalemia, and hypomagnesemia that required correction on multiple occasions. Hypomagnesemia was resolved after nine doses of magnesium sulfate IV as well as oral magnesium oxide. Sodium chloride replacements were administered at 3%. Pancreatitis resolved on day two of admission, after which a diabetic diet of 2,200 kcal/day was started. Insulin was transitioned to the subcutaneous route at this time as well. She remained hospitalized for a total of nine days, whereupon electrolytes normalized, renal function improved, and blood glucose levels stabilized. The patient was discharged following extensive education and a treatment plan including a 2,200 kcal/day diet, an insulin regimen, and follow-up with a pediatrician and a pediatric endocrinologist. Upon discharge, the patient was started on olezarsen for control of triglyceride levels in the setting of familial hypertriglyceridemia (off-label use, as current approval is for use in adult patients), which itself was diagnosed on genetic testing. Her status two years after the initial diagnosis of diabetes and HTG was reported as well-controlled type 1 DM with an HgA1c of 5.5%, adequate growth, and normalized lipid levels.

The patient had a traditional DKA presentation, but unexpectedly, lipemic blood samples raised suspicion for other conditions such as primary or secondary hypertriglyceridemia. This emphasizes the importance of thorough past medical and family history taking to better manage and prevent future episodes of acute pancreatitis and DKA in a diabetic patient.

## Discussion

Acute pancreatitis often has a non-specific presentation in the pediatric population, making the process of establishing its etiology less clear-cut when compared to the adult setting [4]. In this case, the patient presented with HTG and de novo DM, a very common finding in adult and pediatric patients [3]. Yet the incidence of hypertriglyceridemia-induced pancreatitis has not been well-defined in children compared to adults. In adults, HTG-induced AP is the third most common cause after gallstones and alcohol. While in children, there is less literature pertaining to its incidence [5]. 

Serum TG is highly determined by both genetic and environmental elements. Of these, it is believed that factors such as diet, alcohol intake, insulin deficiency/resistance, physical activity, adipose function, and others add to the impact that any genetic influence may have [6]. Serum TG levels >1000 mg/dL have been closely associated with AP, but the exact level after which AP occurs has not been established and often varies [7]. A lipemic (milky) appearance of serum or plasma is an immediate visual cue that suggests marked hypertriglyceridemia, often with triglyceride levels >1000 mg/dL. This can serve as a bedside indicator of a metabolic derangement beyond hyperglycemia and ketosis, alerting clinicians to the possibility of hypertriglyceridemia-induced complications, including AP. Patients diagnosed with DKA with hypertriglyceridemia should undergo genetic testing to rule out the possibility of familial causes and potential mutations in the LPL gene. In DKA patients, hypertriglyceridemia can lead to longer-lasting acidosis and other complications. There is also the possibility of spurious laboratory results showing pseudo-hyponatremia or abnormally low bicarbonate levels [8]. True electrolyte imbalances can also occur with marked hypertriglyceridemia, and results should be evaluated and assessed with caution to prevent incorrect therapies from being administered [9].

There are several theories as to the pathophysiology behind HTG-induced acute pancreatitis. These often concur that hydrolysis of triglycerides by pancreatic lipase to free fatty acids leads to damage to acinar and endothelial cells, as well as ischemia and hyperviscosity due to increased chylomicrons. This pathway is enhanced in the insulin-deficient state of DKA [5,10].

Another marker that can be used to assess the possibility and severity of DKA is the neutrophil-to-lymphocyte ratio (NLR). A higher NLR correlates with DKA severity in new-onset type 1 DM; however, its value for detecting concomitant pancreatitis is uncertain, and it should not be used in isolation [11].

HTG and diabetes mellitus are often intertwined. There is a known association between these and elevated TG and cholesterol that improve with insulin therapy. The simultaneous occurrence of DKA with HTG-induced pancreatitis in a single patient is uncommon, with reports showing increased inpatient mortality (adjusted odds ratio (aOR) 2.8), acute kidney injury, sepsis, and longer length of stay when compared to those with pancreatitis without HTG or DKA [12]. The management of HTG-induced acute pancreatitis primarily involves reducing the serum triglyceride levels. This can be achieved by dietary modification, lipid-lowering medications, or plasmapheresis. Intravenous insulin can also be used for the treatment of severe HTG, which also happens to be the management of DKA. However, in the context of HTG-induced pancreatitis, insulin resistance can be more severe, necessitating higher doses of insulin and very close glucose monitoring. Some patients may require administration of insulin at 0.2 IU/kg to treat both elevated triglyceride levels and DKA [12-14]. In our patient, the insulin was administered at 0.1 IU/kg with adequate improvement in both DKA and hypertriglyceridemia.

## Conclusions

This case aims to highlight the importance of considering hypertriglyceridemia as an inciting factor for pancreatitis and subsequent diabetic ketoacidosis in a pediatric patient, even in the absence of prior metabolic disease. The presence of lipemic serum in a blood sample should prompt clinicians to investigate for severe hypertriglyceridemia, as early recognition can guide appropriate management and prevent complications such as pancreatitis, electrolyte imbalances, multiorgan dysfunction, or, as in this case, diabetic ketoacidosis. Prompt initiation of insulin therapy, aggressive fluid resuscitation, and close monitoring of electrolytes are still first-line treatments. This case affirms the value of thorough family history, dietary assessment, and long-term follow-up to identify possible genetic disorders and reduce recurrence risk.
